# A systematic literature review on strategies to avoid look-alike errors of labels

**DOI:** 10.1007/s00228-018-2471-z

**Published:** 2018-05-12

**Authors:** Karin H. M. Larmené-Beld, E. Kim Alting, Katja Taxis

**Affiliations:** 1 Department of Clinical Pharmacy, Isala Hospital, Dokter van Heesweg 2, 8025 AB Zwolle, The Netherlands; 2 Faculty of Mathematics and Natural Sciences, PharmacoTherapy, -Epidemiology and -Economics, Groningen Research Institute of Pharmacy, University of Groningen, Groningen, The Netherlands

**Keywords:** Look-alike, Label, Tall Man, Color coding, Medication

## Abstract

**Purpose:**

Unclear labeling has been recognized as an important cause of look-alike medication errors. The aim of this literature review is to systematically evaluate the current evidence on strategies to minimize medication errors due to look-alike labels.

**Methods:**

A literature search of PubMed and EMBASE for all available years was performed independently by two reviewers. Original studies assessing strategies to minimize medication errors due to look-alike labels focusing on readability of labels by health professionals or consumers were included. Data were analyzed descriptively due to the variability of study methods.

**Results:**

Sixteen studies were included. Thirteen studies were performed in a laboratory and three in a healthcare setting. Eleven studies evaluated Tall Man lettering, i.e., capitalizing parts of the drug name, two color-coding, and three studies other strategies. In six studies, lower error rates were found for the Tall Man letter strategy; one showed significantly higher error rates. Effects of Tall Man lettering on response time were more varied. A study in the hospital setting did not show an effect on the potential look-alike sound-alike error rate by introducing Tall Man lettering. Color-coding had no effect on the prevention of syringe-swaps in one study.

**Conclusions:**

Studies performed in laboratory settings showed that Tall Man lettering contributed to a better readability of medication labels. Only few studies evaluated other strategies such as color-coding. More evidence, especially from real-life setting is needed to support safe labeling strategies.

**Electronic supplementary material:**

The online version of this article (10.1007/s00228-018-2471-z) contains supplementary material, which is available to authorized users.

## Introduction

Good labeling of medication is an important aspect of medication safety. The American Food and Drug Administration (FDA) estimated that 20% of medication errors may be attributed to confusing packaging and poor labeling; others suggested even higher rates [1, 2]. Commonly, look-alike labels due to similar drug names, e.g. ceftazidime—ceftriaxone or other readability issues are a cause for these errors. The clarity of labels on the primary containers of medications, also called primary labels, is particularly important for healthcare professionals. Primary labels, e.g., on vials, ampoules, syringes, or infusion bags are used in the step of medication administration to the patient. Misreading labels resulting in the administration of the wrong drug can have serious consequences for patients [3–5].

Various measures have been suggested to enhance the readability of labels and reduce errors due to look-alike labels [3, 4]. A technical solution is the use of a closed-loop system with barcode technology. But this is currently not widely implemented. Furthermore, in emergency situations, there may not be sufficient time to use barcode systems. Therefore, readability of the labels remains important. But internationally, there is no consensus about the content and form of labels. Guidelines of the FDA and European Medicine Agency (EMA) do not give conclusive advice on how to prevent look-alike errors [5, 6]. Strategies such as Tall Man lettering and color-coding are seen as potential solutions. Tall Man lettering aims to maximize the difference between two similar drug names by capitalizing part of the drug names [7]. This could avoid mixing up two confusing drug names. Several organizations endorsed Tall Man lettering including the Joint Commission and the Institute for Safe Medication Practices (ISMP) [6–8]. Color-coded labels are used in anesthesia to distinguish between different substance classes as described in an international standard (ISO 26825) [9]. Also, best practices are available with design features to improve the design of labels and claim that this would improve patient safety [10]. A number of systematic reviews have addressed the related issue of sound-alike drug names, i.e., the problem of phonetic similarity of drug names [11, 12]. Another study addressed all types of dispensing errors [13]. Some of the evidence about Tall Man lettering has been summarized in an Editorial [14]. But no recent systematic review has addressed the question how medication labels, in particular primary labels on syringes, ampoules, or infusion bags, should look like to prevent errors. Therefore, the aim of this literature review was to systematically evaluate the current evidence for strategies to minimize medication errors due to look-alike labels.

## Literature search method

### Search strategy

This systematic review focused on primary labels on medication containers, e.g., syringes, ampoules, or infusion bags regardless whether they were produced by the industry, the hospital pharmacy department, or on the wards. The literature search was conducted on 14 September 2015 for all available years in PubMed and EMBASE following PRISMA guidelines [15]. We used the MeSH index terms ‘medication error’ and ‘drug labeling’ in combination with the free index terms ‘barcoding’ or ‘sound alike’ or ‘look alike’ or ‘text enhancement’ or ‘enhanced text’ or ‘drug name confusion’ or ‘color-coding’ mentioned in the abstract or title of the study. To prevent missing possible studies about look-alikes, the term sound-alike was added as a search term as these terms are often used as combinations. In addition, the list of references of all included studies and review articles were screened to identify additional references. An example of the search strategy is added as Appendix.

### Inclusion criteria

Original studies assessing strategies to minimize medication errors due to look-alike labels focusing on readability of the primary labels by healthcare professionals or consumers were included. Studies had to report a quantitative outcome related directly or indirectly to medication errors. There were no restrictions on the study design or study setting (e.g., hospital, community pharmacy, laboratory) or the origin of the label (e.g., industry, pharmacy, ward).

### Exclusion criteria

Studies written in other languages than English were excluded. Studies were also excluded if they involved case studies or causes of medication errors other than look-alike errors, such as sound-alike errors or failing communication between doctors and nurses.

Two reviewers (KLB, EKA) screened the titles and abstracts of the retrieved records independently [16]. Full texts of all potentially eligible records were also examined independently by the two reviewers. Disagreements were resolved by consensus.

### Definition medication error

Medication errors were defined as a discrepancy between the drug therapy received by a patient and the drug therapy intended by the prescriber [1]. This was extrapolated to the setting of the labels by any discrepancy in readability by intended variation and distracting variation in the label by any strategy, e.g., color, Tall man lettering.

### Data extraction

The following data were extracted using an Excel spreadsheet: first author’s surname, publication year, country of origin, setting, participants, sample size, type of strategy to prevent look-alike errors, tested product, and drug names tested. We extracted all outcomes directly and indirectly related to medication errors. The outcomes of the studies were extracted, with no manipulation; e.g., the error rates as reported in the study were extracted without adjustment. Extraction was done by EKA and verified by KLB. Disagreements were resolved by discussion between the two reviewers and a third reviewer (KT) until consensus was reached.

### Data synthesis and analysis

The studies were grouped based on the type of intervention tested (e.g., Tall Man lettering, color) and the type of outcome. Error (rate) and response times were used in the majority of the included studies. Overall error rates were included in the results of the review because not all included studies reported on different subtypes of error. For one study, the authors were contacted for missing information but without response. A note has been made to the results. Due to the high variability of the design of the included studies, it was not possible to perform a meta-analysis. Therefore, data were analyzed descriptively.

## Results

**Fig. 1:**
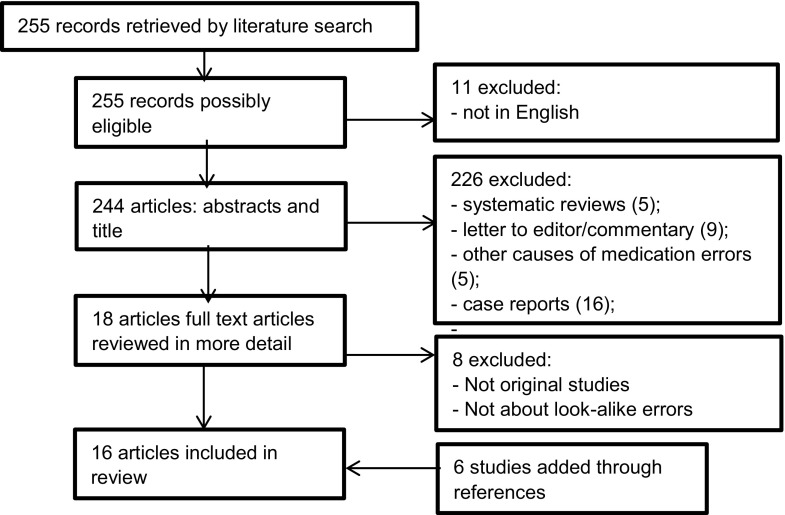
Flow chart summarizing study selection

The literature search resulted in a total of 255 studies. The full text of 18 articles were reviewed and 16 articles were included in the systematic review (Fig. 1).

The main characteristics of the included studies are described in Table 1. The majority of studies were conducted in the UK and USA, namely five each [18–20, 22, 24–27, 31, 32]. The remaining six studies were performed in Canada, China, India, Ireland, and Norway [17, 21, 23, 28–30]. Of the sixteen studies, half used healthcare professionals for the tests and half used non-healthcare professionals. All studies were controlled laboratory experiments, except three studies which were conducted in a hospital environment. The strategies which were tested were Tall Man lettering, color-coding, variations in the background of the label, and the use of symbols.

**Table 1:** Overview of included studies

First author	Publication year	Country	Setting	Method of testing	Participants	Strategy
Fasting [17]	2000	Norway	Hospital	Checklist on anesthetic chart	Anesthesiologists	Color-coding
Filik [18]	2004	UK	Laboratory	Mock drug packs with similar names	Non-healthcare professionals	Tall Man lettering
Filik [19]	2006	UK	Laboratory	Pairs of generic drug names	Non-healthcare professionals	Tall Man lettering
					Non-healthcare professionals	Tall Man + color
Gabriele [20]	2006	USA	Laboratory	Look-alike drug names	Acute care hospital nurses	Typographic including Tall Man lettering
Momtahan [21]	2008	Canada	Laboratory	Available ampoules and vials in hospital setting	Registered nurses	Contrasting background
Schell [22]	2009	USA	Laboratory	Confusable drug name pairs	Non-healthcare professionals	Tall Man lettering
Shannon [23]	2009	Ireland	Hospital	Questionnaire	Physicians	Color-coding
Filik [24]	2010	UK	Laboratory	Pairs of similar drug names	Young and older adults	Tall Man lettering
				Confusable drug name pairs	Healthcare professionals	Tall Man lettering
Darker [25]	2011	UK	Laboratory	Confusable drug name pairs	Healthcare professionals	Tall Man lettering
Cardarelli [26]	2011	USA	Laboratory	Symbols for medication indications	Older patients	Symbol use (TCOM system)
Irwin [27]	2013	UK	Laboratory	Target drug names	Non-pharmacists and pharmacy staff	Typographic including Tall Man lettering
Or [28]	2014	China	Laboratory	Pairs of similar drug names	Non-healthcare professionals and registered nurses	Typographic including Tall Man lettering
Or [29]	2014	China	Laboratory	Pairs of similar drug names	Non-healthcare professionals	Tall Man lettering
Gupta [30]	2015	India	Laboratory	Ampoules in hospital	Physicians (residents)	Contrasting background
Zhong [31]	2015	USA	Hospital	Look-alike and sound-alike drug pairs	Clinical pharmacists, physicians	Tall Man lettering
DeHenau [32]	2016	USA	Laboratory	Comparable drug labels	Healthcare professionals and laypeople	Tall Man lettering

*TCOM* tachygraphic color organized medication

The general study design and the study methods varied widely between studies. This included the number of participants, the number of experiments, the type of medication names, and the test conditions. The most important details are summarized per study in Tables 1 and 2 and in Table 3 and 4 in the Appendix.

**Table 2:** Results of the studies testing Tall Man lettering for the outcomes error rate, items correctly selected and other outcomes

Ref. no.	Testing conditions	Method	Sample Size (*n* = number of participants)	Main Results		
				Tall Man	Non-Tall Man	*p* value
				Error rate		
18	Tall Man vs. lowercase lettering	20 mock drug packs with 1 pair of similar drug names	20 arrays (*n* = 20)	3%	7.8%	*p* < 0.05
19	Tall Man vs. lowercase black	60 pairs of generic, similar drug names	12 experimental blocks (*n* = 28)	7.8%	8.9%	Letter style: *p* < 0.05
	Tall Man vs. lowercase color			7.7%	8.5%	Color: *ns*
24	Same names Tall Man vs. lowercase	80 pairs of similar and same drug names	80 trials (*n* = 56)	22.3%	19.6%	Similarity: *p* < 0.05
	Different names Tall Man vs. lowercase			11.1%	17.2%	Letter style: *p* < 0.001
24	Tall Man vs. lowercase (target absent)	20 confusable drug name pairs	160 trials (*n* = 127)	3.1%	4.3%	Letter style: *p* < 0.05
	Tall Man vs. lowercase (target present)			4.3%	4.5%	
25	Lowercase vs.	20 confusable drug name pairs	200 trials (*n* = 144)	–	22%	
	Tall Man wild^*^			16%^#^	–	*All p* < 0.001
	Tall Man mid			16%	–	
	Tall Man CD3			18%	–	
	All uppercase			16%	–	
	Tall Man CD3 vs.			18%	–	*p* < 0.05
	Tall Man mid			16%	–	*p* < 0.01
	All uppercase			16%	–	
22	Overall (all types) No enhancement Colored text enhancement Case-based enhancement	80 confusable drug name pairs	240 trials (*n* = 102)	6.1% - - 7.5%	- 5.4% 5.3% -	*p* < 0.05
29	Engineering students (no pharmacy background)	28 confusable drug name pairs and 28 identical drug name pairs	336 trials (*n* = 40)			
	Tall Man vs.			8.0%^#^		*p* < 0.001
	- Lowercase				24.2%	
	- Boldface				5.1%	
	- Boldface plus Tall Man				3.3%	
	- Color				4.8%	
	- Contrast				5.8%	
	Pharmacy students		336 trials (n = 40)			
	Tall Man vs.			4.5%^#^		*p* < 0.001
	- Lowercase				9.8%	
	- Boldface				3.3%	
	- Boldface plus Tall Man				2.5%	
	- Color				2.8%	
	- Contrast				2.8%	
		Items correctly selected				
27	Non-pharmacists					
	- Tall Man vs. lowercase with time pressure	50 target drug names	100 trials (*n* = 60)	48.0%	48.1%	Time pressure: *ns*
	- Tall Man vs. lowercase without time pressure		100 trials (*n* = 28)	49.3%	49.5%	Tall Man: *ns*
	Pharmacist					
	- Tall Man vs. lowercase with time pressure	50 target drug names		49.6%	49.3%	Time pressure: *ns*
	- Tall Man vs. lowercase without time pressure			49.7%	49.6%	Tall Man: *ns*
		Other				
31	Number of potential errors 11 look-alike/sound-alike drug pairs	1.676.7000 patient charts (0–20 years)	Hospitalizations (*n* = 1.676.700)	0.50–0.75 per 1000 hospitalizations	0.64–1.44	*ns*
32	% of change detections Tall Man format vs. traditional format	16 drug labels	16 clinical trials (*n* = 80)	95.1%	85.9%	*p* < 0.0001

^*^The Wild Tall Man rule used examples of how Tall Man lettering has been implemented from projects. The Mid Tall Man rule capitalizes the first letters at either end that differ, along with all letters occurring between them (cefIXime, cefOTAXime, cefTAZIDime, cefUROXime). The CD3 Tall man rule as Mid Tall Man, but capitalizes a maximum of three letters (cefiXime, cefOTAxamine, cefTAZidime, cefUROxime)

^#^For better comparison, these percentages have been calculated based on the results presented in the paper

### Tall man lettering

Eleven studies evaluated the use of Tall Man lettering (Tables 1 and 2 and Table 3 (Appendix)) [18–20, 22, 24, 25, 27–29, 31, 32]. A wide range of different drug names were tested. Most studies tested pairs of similar drug names, but two studies also tested Tall Man lettering on the same name, i.e., presenting the drug name once in Tall Man lettering and once in non-Tall Man lettering [19, 24].

Some studies tested variations of Tall Man lettering, e.g., capitalizing different parts of the drug name [25, 29]. Some studies tested additional conditions such as with and without time pressure or previous knowledge about the purpose of Tall Man lettering [19, 27]. The most common outcomes tested were error rate and response time. Some studies assessed subtypes of errors, such as commission and omission errors, others only reported overall error rate. In the tables, only overall error rates are reported. One study tested the number of items correctly selected. Other outcomes included eye movements, hospital admissions, and change detection (Table 2).

#### Error rate

Medication error rates ranged from 3 to 22% for Tall Man lettering and from 3 to 24% for non-Tall Man lettering. Six out of seven experiments showed that participants made significantly fewer errors when the drug names contained Tall Man letters, than when the drug names were displayed in lowercase letters [18, 19, 24, 25, 29]. From these six experiments three were performed by healthcare professionals [24, 25, 29] and three were performed by younger and older adults [24], university students [22] and engineering students [29]. One study in non-healthcare professionals found a significantly higher error rate with Tall Man lettering. Only one study was performed in a hospital setting and resulted in no beneficial effect for Tall Man lettering to reduce potential look-alike sound-alike error rates [31]. Several methodological limitations may contribute to these results [14, 31].

#### Response time

Response times ranged from 1.2 to 31 s for Tall Man lettering and from 1.3 to 47 s for non-Tall Man lettering. In three out of nine studies, response time was significantly shorter for Tall Man lettering compared to non-Tall Man lettering [19, 24, 32]; in four studies, there was no difference [18, 19, 27]; and in two studies, response time was significantly longer for Tall Man lettering [24, 28]. One study found that when participants did not know about the purpose of Tall Man lettering, the response times were similar for lowercase and Tall Man lettering. But when told, the response times were shorter for Tall Man lettering [19].

#### Variation in Tall Man lettering

Other text enhancement methods (larger lowercase, boldface, and colored lettering) had also lower medication error rates and shorter response times compared to the lowercase condition [28, 29]. The use of boldface plus Tall Man lettering performed best [29].

#### Other outcome measures

Eye movement experiments (eye tracking system to determine fixation points) showed that participants spent less time fixating on the “distractor drug packs” (wrong pack) with Tall Man letters than on “distractor drug packs” with lowercase names (1.42 vs. 1.90 s, *p* < 0.005). Also the number of fixations was fewer for the drug packs with Tall Man letters than for the drug packs with lowercase lettering (4.6 fixations vs. 5.6 fixations, *p* < 0.05) [18].

### Other strategies

Two studies tested color-coding (Table 4, Appendix) [17, 23]. One study showed no reduction in incidence and severity of drug errors after introducing color-coded syringe labels [17]. A questionnaire-based study reported that physicians still experienced medication errors and near misses after introduction of a color-coded system [23]. Other studies performed experiments that focused on contrasting backgrounds on ampoules. The time it took the participants to identify the information on the existing labels (text directly printed on glass or on a clear label) was significantly longer than for the new white labels. The correct reading score was higher for the ampoules with a white label than for the ampoules with text directly printed on glass or on a clear label [21, 30].

The study on symbol use (Table 4, Appendix) showed that the addition of symbols on labels improved the percentage correctly identified medications significantly when reading labels at a 2-ft distance [26].

## Discussion

In our systematic literature review, we found evidence from laboratory-based studies that Tall Man lettering contributes to a better readability of medication labels. There are only few studies evaluating color-coding or other strategies such as the use of symbols.

Almost all studies on Tall Man lettering showed a lower error rate, except the study by Schell [22], but examining details of the study suggests that there may have been a ceiling effect of accuracy, i.e., a very low error rate and a small sample size for one of the experiments questioning statistical testing which may explain the negative findings of this study. Most studies also assessed response time. This measure provides general information on how people may process information on the label under time pressure, e.g., due to high workload. In healthcare, high workload has been linked to declines in checking accuracy and a decrease in visual fixations resulting in error-producing conditions [33]. Studies investigating the effects of Tall Man lettering on response time are less conclusive. Only three out of nine experiments showed significant results in favor of Tall Man lettering [19, 24, 32]. Interestingly, Filik et al. showed that response time depended whether or not participants knew about the purpose of Tall Man lettering [19]. This may suggest that training may be required for optimal use of the Tall Man lettering strategy. Of note, other studies do not explicitly state whether or not participants knew about the purpose of the study. The results of Or et al. suggest that combining Tall Man lettering and greater stroke widths increases the salience of highlighted letters decreasing the difficulty of the visual search and detection which improves name differentiation [29]. In summary, the laboratory experiments show promising results for Tall Man letter strategies to make similar names less confusable perceptually and can increase attention to high-risk drug names. But details of which type of Tall Man lettering works best, for example, which part of the word should be capitalized, cannot be derived because the used pairs of drug names in the studies were different and also which specific letters were written in Tall Man lettering were different between the studies [19, 24, 25].

In a very interesting study, Schroeder et al. found a significant relationship between drug name confusion rates in laboratory-based memory and perception tests and error rates in community pharmacy practice. In short, they were able to predict error rates based on laboratory experiments. In analogy, Tall Man lettering, could contribute to a higher level of medication safety. But it remains challenging to assess the specific contribution of one factor, like Tall Man lettering, because the administration of medication is a process in which many factors play a role [34, 35]. By and large, real-world data are lacking. A recent time series analysis in US hospitals showed no reduction in look-alike errors after the implementation of Tall Man lettering [31]. These negative results may be due to a lack of implementation of the strategy in the hospitals. It remained unknown to what extent and when (if at all) the hospitals implemented Tall Man lettering and for which name pairs [31]. This may suggest that additional translational research is needed to identify the implementation measures needed to improve medication safety in practice using Tall Man lettering [36]. Such questions need to be answered, alongside well-conducted studies in practice to investigate the benefit or otherwise of Tall Man lettering for medication safety. This is urgent, as Tall Man lettering seems widely embraced as an error reduction strategy [7, 37, 38].

The evidence for a color-coding system is scarce. The little evidence there is suggests that color-coding does not reduce the risk of look-alike drug errors. A number of other arguments have been raised against the use of color-coding. There are far more look-alike drugs or drug groups than there are colors which could be used. Furthermore, the prevalence of congenital color vision deficiency is about 8% for men and 0.4% for women in the general population [39]. Most importantly, evidence suggests that healthcare professionals will rely solely on the color of the labels, and not read the labels at all [40, 41]. Anecdotal evidence suggests problems implementing the color-coding system in practice [42]. Despite the lack of evidence, there is an international standard recommending color-coding in anesthesia and it seems to be the most commonly used strategy for label enhancement in anesthesia at the moment, used in multiple countries around the world, including the UK, Australia, and New Zealand [9, 43, 44].

A number of important methodological issues of the included studies have to be considered. First, as highlighted above, most experiments were conducted in a controlled laboratory environment rather than in a hospital. This restricts the generalizability of the findings to a busy ward environment with numerous other factors (workload, stress, noise, lightning) having an impact on the performance of users [13]. Second, as in other medication error research, no standard definition of what constituted an ‘error’ was used [45, 46]. For example, some authors defined ‘error’ as the overall errors that were made; others used different categories, like omission and commission errors. Therefore, error rates may not be comparable between studies and this may explain that the error rates ranged between 3 and 24%. These and other methodological differences, such as the wide range of different drug names tested, between studies made it impossible to carry out a formal meta-analysis of the data. Studies using appropriate methods and well-defined outcome measures are needed to evaluate the effects of the different label enhancement methods in practice. Such studies should be carried out before widespread implementation of label enhancement strategies.

Our study has a number of limitations which need to be considered. First, only two databases were used for the literature search as we expected that these databases contained the relevant literature. We also did not carry out a formal search of gray literature. All references of the included studies were analyzed to check for possible related studies. This resulted in six more studies which may mean that our electronic search was not as comprehensive as we intended. Nevertheless, we are confident that the electronic search combined with the hand search was successful in locating the relevant literature. Second, we only included English publications, so we may have missed studies published in other languages. Finally, we did not perform a detailed assessment of the quality of the studies as currently available instruments were not suitable for the laboratory-based studies included in our review [47].

Safer labeling of medication is only one aspect of preventing medication errors due to mixing up medications. Labeling needs to be part of a multifaceted approach involving many different aspects of the medication use process. This includes the selection of the non-sound-alike/look-alike generic and brand names during the drug development process. In practice, this concerns procedures such as double-checking of medications before administration [48], the use of technological solutions such as bar-coded drug administration [49] and consumer education [11]. But, given the scale of daily use of labels to identify medications in practice by healthcare professionals and patients, it is surprising that the fundamental question of what a medication label should look like cannot be answered adequately [5, 6].

## Conclusion

Laboratory studies show that Tall Man lettering contributes to reduced error rates probably due to a better readability of medication labels, but evaluations in real-life setting are needed to strengthen this conclusion. There is little evidence supporting color-coding and few other methods such as symbols have been tested.

## Electronic supplementary material

ESM 1 (DOCX 13 kb)

ESM 2 (DOCX 28 kb)
