# Supplementary material for: A systematic literature review on strategies to avoid look-alike errors of labels
Source: Eur J Clin Pharmacol. 2018 May 12;74(8):985–93. doi: 10.1007/s00228-018-2471-z (PMC6061459; doi:10.1007/s00228-018-2471-z)
Supplement: Supplementary file 1 — (DOCX 13 kb) [file 228_2018_2471_MOESM1_ESM.docx]

Search strategy; A systematic literature review on strategies to avoid look-alike errors in hospital practice

#11 #1 AND #10

#10 #2 OR #3 OR #4 OR #5 OR #6 OR #7 OR #8

#9 *'color coding':ab,ti*

#8 *'drug name confusion':ab,ti*

#7 *'enhanced text':ab,ti*

#6 *'text enhancement':ab,ti*

#5 *'look alike':ab,ti*

#4 *'sound alike':ab,ti*

#3 *barcoding:ab,ti*

#2 *'drug labeling'/exp/mj*

#1 *'medication error'/exp/mj*
