# Supplementary material for: A systematic literature review on strategies to avoid look-alike errors of labels
Source: Eur J Clin Pharmacol. 2018 May 12;74(8):985–93. doi: 10.1007/s00228-018-2471-z (PMC6061459; doi:10.1007/s00228-018-2471-z)
Supplement: Supplementary file 2 — (DOCX 28 kb) [file 228_2018_2471_MOESM2_ESM.docx]

Table 3: Results of the studies testing Tall Man lettering for the outcome response time

| Ref. no. | Testing conditions | Method | Sample Size  (n=number of participants) | Main Results | | |
| --- | --- | --- | --- | --- | --- | --- |
|  |  |  |  | *Tall Man* | *Non-Tall Man* | *p-value* |
| 19 | *Without knowledge of purpose Tall Man letters*  Same names Tall Man vs. lowercase  Different names Tall Man vs. lowercase  *Knowledge of purpose Tall Man letters*  Same names Tall Man vs. lowercase  Different names Tall Man vs. lowercase | 80 pairs of similar and same generic drug names | 80 trials (n=40) | 2223 ms  1713 ms  1451 ms  1320 ms | 2214 ms  1752 ms  1571 ms  1521 ms | Similarity: *p*<0.0005  Letter style: *ns*  Similarity: *p*<0.005  Letter style: *p*<0.01 |
| 24 | Same names Tall Man vs. lowercase  Different names Tall Man vs. lowercase  Tall Man vs. lowercase (target absent)^*^  Tall Man vs. lowercase (target present) | 80 pairs of similar and same drug names  20 confusable drug name pairs | 80 trials (n=56)  160 trials (n=127) | 1366 ms  1245 ms  2304 ms  1699 ms | 1295 ms  1297 ms  2246 ms  1635 ms | Similarity: *p*<0.05  Letter style: *p*<0.005  Target: *p*<0.001  Letter style: *p*<0.001 |
| 27 | Reaction time *non pharmacists*  - Tall Man vs. lowercase with time pressure  - Tall Man vs. lowercase without time pressure  Reaction time *pharmacists*  -Tall Man vs. lowercase with time pressure  -Tall Man vs. lowercase without time pressure | 50 similar, target drug names | 100 trials (n=60)  100 trials (n=28) | 8.6s  9.4s  8.7s  10.2s | 8.6s  9.1s  8.4s  9.8s | Time pressure: *ns*  Tall Man: *ns*  Time pressure: *ns*  Tall Man: *ns* |
| 18 | Search time Tall Man vs. lowercase | 20 mock drug packs with 1 pair of similar drug names | 20 arrays (n=20) | 10.11s | 9.82s | *ns* |
| 32 | Mean estimates of change detection time Tall Man vs. lowercase^#^  - 16 Nurses  - 24 Other healthcare providers  - Laypeople | 16 drug labels | 16 clinical trials (n=80) | 27s  31s  27s | 47s  41s  33s | *p*<0.0001  *p<0.017*  *p*<0.015 |
| 28 | Mean response time Tall Man vs.  - Lowercase  - Boldface  - Larger lowercase  - Red lettering | 120 confusable drug name pairs | 120 trials (n=60) | 2.1s | 2.0s  2.0s  1.8s  1.9s | *p*<0.05 |

^*^ The target was one of two names of a confusable drug pair. The one presented to the participants in advance, was the target to search for (whether present or absent).

^#^ The mean estimates of change detection time have been estimated, because the exact data was not given.

*ms = miliseconds s = seconds ns = not significant*

Table 4: Results of studies testing other methods of label enhancement.

| Ref. no. | Testing conditions | Outcomes tested | Method | Sample size | Results |
| --- | --- | --- | --- | --- | --- |
| 17 | Color-coding | Drug errors before and after implementation colored label  Type of drug errors before and after colored label implementation  - Ampoule swap  - Syringe swap  - Other causes wrong drug  - Wrong dose | Checklist on anesthetic chart | 28971 and 26455 cases | 0.14% vs. 0.087%, *ns*  0.028 vs 0.004%, p=0.04  0.055 vs 0.045%  0.014 vs 0.015%  0.041 vs 0.023% |
| 23 | Color-coding | Percentage of answers   - Incorrect medication administered - Near miss - Color has influence on judgment | Questionnaire | 30 questionnaires | 23%  53%  50% |
| 26 | Symbol use | Percentage drugs correctly matched with indication   - Old label, in front of participant - Old label, 2-feet distance - New label, in front of participant - New label, 2-feet distance | 19 symbols for medication indications | 100 participants | 88.5%  81.1%  92.3%, *ns*  88.6%, *p<0.001* |
| 30 | Contrasting background | Mean reading time original vs. modified ampoule | 2 ampoules (1 text on glass or clear substrate and 1 text on white substrate) | Group A and B each 54 participants | 11.64 vs. 9.48  *p*<0.01 |
| 21 | Contrasting background | Accuracy of identifying ‘route of administration’ ampoule D3^*^: on glass vs. white label  Mean reaction time of identifying information on ampoule D3 (on glass vs. white label)  Drug concentration:  Generic Name:  Route of administration: | 6 ampoules (3 text on glass or clear substrate and 3 text on white substrate) | 18 trials (n=24) | 50.0% vs. 79.2%  *p<0.05*  7.8s vs. 5.8s^#^  4.9s vs. 3.7s  7.3s vs. 5.2s  *p<0.0001* |

^*^Ampoule D3 contains paraldehyde.

^#^ The mean reaction times have been estimated, because the exact data was not given. *s = seconds ns = not significant*
